# Can Ferritin Levels Predict the Severity of Illness in Patients With COVID-19?

**DOI:** 10.7759/cureus.12832

**Published:** 2021-01-21

**Authors:** Firdevs Tugba Bozkurt, Mehmet Tercan, Gulcin Patmano, Tugba Bingol Tanrıverdi, Huseyin Avni Demir, Ugur Fahri Yurekli

**Affiliations:** 1 Intensive Care Unit, University of Health Sciences, Şanlıurfa Mehmet Akif İnan Research and Training Hospital, Şanlıurfa, TUR; 2 Department of Anesthesiology and Reanimation, University of Health Sciences, Şanlıurfa Mehmet Akif İnan Research and Training Hospital, Şanlıurfa, TUR; 3 Department of Emergency Medicine, University of Health Sciences, Şanlıurfa Mehmet Akif İnan Research and Training Hospital, Şanlıurfa, TUR; 4 Department of Biochemistry, University of Health Sciences, Şanlıurfa Mehmet Akif İnan Research and Training Hospital, Şanlıurfa, TUR

**Keywords:** pandemic, coronavirus, ferritin, disease severity

## Abstract

Objectives: This study aimed to investigate whether ferritin level can predict the severity of coronavirus disease 2019 (COVID-19).

Background: The COVID-19 pandemic has been challenging for both patients and caregivers. Many laboratory markers have been used to better understand the causes of poor outcomes and to improve the management of COVID-19 patients.

Methods: A total of 93 patients who had a positive polymerase chain reaction test result for severe acute respiratory syndrome coronavirus 2 (SARS-CoV-2) were included in this study. Demographic features, comorbidities, clinical and laboratory findings were obtained from the hospital database retrospectively. Patients were divided into two groups according to the disease severity as follows: mild group (n = 70) and severe group (n = 23).

Results: The median age of the study population was 42.5 (28.3-62.8) with 69.9% male patients. Patients in the severe group were significantly older and showed a higher frequency of hypertension, diabetes mellitus, coronary artery disease, and heart failure in comparison with those in the mild group. In addition, gamma-glutamyl transferase, C-reactive protein, ferritin, interleukin-6, procalcitonin, and neutrophil to lymphocyte ratio were higher whereas albumin level was lower in patients in the severe group. Linear regression analysis demonstrated that ferritin level was the only significant predictor of disease severity (β = 0.487, t = 2.993, p = 0.004). In receiver operator characteristics curve analysis, ferritin level ≥264.5 ng/mL predicted severe COVID-19 with a sensitivity of 73.9% and specificity of 94.2%.

Conclusion: Early analysis of ferritin levels in patients with COVID-19 might effectively predict the disease severity.

## Introduction

Severe acute respiratory syndrome coronavirus-2 (SARS-CoV-2), which causes coronavirus disease 2019 (COVID-19), has rapidly spread all over the world and infected millions of individuals. It has been found that the virus affects host human cells by binding to the angiotensin-converting enzyme 2 (ACE-II) receptor [1]. Despite initial findings that showed that SARS-CoV-2 causes a respiratory tract infection, current data have proved its systemic effects on all the body systems [2,3]. Elderly individuals and those with several comorbidities are at greater risk of mortality and morbidity compared with younger individuals. However, the fact that younger individuals without major comorbidities can also develop potentially mortal complications, such as fulminant myocarditis and disseminated intravascular coagulopathy, should be remembered [4,5]. The disease may trigger a broad inflammatory process and cause sepsis, septic shock, and multiple organ dysfunction syndrome, which requires mechanical ventilatory support [6]. The exact underlying pathophysiological mechanisms remain unclear and no reliable marker is yet available to predict the severity and progression of the disease.

Many laboratory markers were investigated in previous studies to better understand the pathogenesis of the disease and to assess how these markers play a role during the COVID-19 process [7]. In addition, recent data have indicated that patients with COVID-19 have decreased hemoglobin levels and elevated levels of ferritin. A study conducted in the United States of America (USA) demonstrated that ferritin levels were pathologically high in 5,700 patients hospitalized for COVID-19 [8]. Anemia accompanying hyperferritinemia is a strong factor for mortality regardless of underlying conditions. Elevated ferritin levels may be predictive of an imminent inflammatory reaction in COVID-19 or be associated with viral spread in the human body and affect iron metabolism [9,10]. In relation, iron is a crucial micronutrient for both human cells and pathogens. To restrain the pathogen’s use of it, the natural immune response may limit iron turnover during infections. However, this mechanism can also lead to anemia, which reduces oxygen delivery to tissues and results in multi-organ failure [10,11]. Therefore, we believe that it is important to determine the association between iron metabolism and progression of COVID-19. The aim of our study is to investigate whether ferritin level can predict the severity of COVID-19.

## Materials and methods

After approval obtained from Harran University Ethics Committee (Grant number: 15/06/2020-11/34) and Ministry of Health (Grant number: 2020-06-01T11_32_48), this study was initiated with a retrospective design that enrolled patients hospitalized with COVID-19 at Mehmet Akif Inan Research and Training Hospital between March 2020 and June 2020. The study was completed in compliance with the World Medical Association Declaration of Helsinki. All patients had a positive polymerase chain reaction (PCR) test result of SARS-CoV-2. Patients aged below 18, with a history of liver malignancy, liver failure, cirrhosis, hepatitis, receiving medical treatment leading to impairment in liver function tests, pregnant women, and individuals with cancer were excluded. The demographic features, comorbidities such as chronic obstructive pulmonary disease (COPD), asthma, chronic renal failure (CRF), diabetes mellitus (DM), hypertension (HT), heart failure (HF), clinical and laboratory findings including white blood cell count (WBC), neutrophil, monocyte, lymphocyte, reticulocyte distribution width (RDW), creatinine, aspartate aminotransferase (AST), alanine aminotransferase (ALT), gamma-glutamyl transferase (GGT), total bilirubin, albumin, c-reactive protein (CRP), ferritin, interleukin-6 (IL-6), and procalcitonin were obtained from the hospital database. Patients were divided into two groups where subjects admitted to ICU were assigned as Group SC and individuals admitted to ward as Group MM. All data were recorded into a standardized datasheet.

Statistical analysis

The distribution of the variables was analyzed using Kolmogorov-Smirnov test. Quantitative data were presented as mean and standard deviation where qualitative data as median (interquartile range (IQR) 25%-75%) values, and also with numbers and percentage. The analysis of the demographic characteristics was conducted using Chi-square test or Fisher’s exact test. The comparison of the laboratory findings was completed by Mann-Whitney U test. A linear regression analysis was used to determine the predictors affecting disease severity. A receiver operating curve (ROC) was performed to find a cut-off value for the possible predictors affecting disease severity, and the sensitivity and specificity of these predictors. All data were analyzed using Statistical Package for Social Sciences (SPSS) version 20 (IBM Corp., Armonk, NY, USA). The significance level was set at p<0.05.

## Results

A total of 93 patients were included in this study. The median age of the study population was 42.5 (28.3-62.8), and 69.9% (65 patients) were male. Table 1 presents the demographic features of the study groups. Patients in the severe group were significantly older and had a higher frequency of hypertension, diabetes mellitus, coronary artery disease, and heart failure compared with patients in the mild group. In addition, the frequency of dyspnea was significantly higher in the severe group than in the mild group. Moreover, hospital stay (days), mechanical ventilation (MV) support rate, and MV duration were significantly higher in the severe group (Table 1). Hematological and biochemical parameters of the study groups are listed in Table 2. Leukocyte count (p = 0.002), neutrophil count (p < 0.05), GGT, CRP, ferritin, IL-6, procalcitonin, and neutrophil to lymphocyte (N/L) ratio were significantly higher, whereas albumin was significantly lower in the severe group than in the mild group.

**Table 1:** Demographic characteristics *p<0.05 a Mann-Whitney U test. b Chi-square test. c Fisher’s exact test. MM, mild-moderate; SC, severe-critical; DM, diabetes mellitus; COPD, chronic obstructive pulmonary disease; CRF, chronic renal failure; HF, heart failure; CAD, coronary artery disease; CT, computerized tomography; MV, mechanical ventilation; ICU, intensive care unit, IQR, interquartile range.

		Group MM (n=70) n (%)	Group SC (n=23) n (%)	P
Gender (Male)		49 (70)	16 (69.6)	0.969^b^
Hypertension		11 (15.7)	10 (43.5)	0.006*^b^
DM		1 (1.4)	4 (17.4)	0.013*^c^
COPD		2 (2.9)	2 (8.7)	0.255^c^
Asthma		10 (14.3)	2 (8.7)	0.724^c^
CRF		-	1 (4.3)	0.247c
HF		-	3 (13)	0.014*^c^
CAD		2 (2.9)	5 (21.7)	0.010*^c^
Symptoms	Cough	36 (51.4)	16 (69.6)	0.129^b^
	Fever	16 (22.9)	9 (39.1)	0.127^b^
	Sore throat	10 (14.3)	−	0.062^c^
	Dyspnea	14 (20)	16 (69.6)	<0.05*^b^
	Headache	4 (5.7)	1 (4.3)	>0.05*^c^
	Fatigue	2 (2.9)	2 (8.7)	0.255^c^
	Myalgia	2 (2.9)	2 (8.7)	0.255^c^
CT results	Typical	14 (20)	15 (65.2)	−
	Possible	7 (10)	4 (17.4)	
	Atypical	11 (15.7)	4 (17.4)	
	Normal	38 (54.3)	−	
Admission	Ward	67 (95.7)	6 (26.1)	<0.05*^c^
	ICU	3 (4.3)	17 (73.9)	
		Median (IQR)	Median (IQR)	
Age (years)		34 (27–50)	63 (55–68)	<0.05*^a^
Hospital stay (days)		6 (5–7.5)	10 (7–15)	<0.05*^a^
MV support		−	14 (60.9)	<0.05*^c^
MV duration		−	2 (0–6)	<0.05*^a^

**Table 2:** Comparison of hematological and biochemical results of the groups *p<0.05 Mann-Whitney U test. MM, mild-moderate; SC, severe-critical; WBC, white blood cell; RDW, reticulocyte distribution width; IQR, interquartile range; AST, aspartate aminotransferase; ALT, alanine aminotransferase; GGT, gamma-glutamyl transferase; CRP, C-reactive protein; IL, interleukin; N/L, neutrophil to lymphocyte.

	Normal ranges	Group MM (n=70) Median (IQR)	Group SC (n=23) Median (IQR)	P
WBC (number/mm^3^)	4000–11000	5820 (4570–6530)	7900 (5180–11170)	0.002*
Neutrophil (number/mm^3^)	2500–8000	3460 (2400–4200)	5460 (3370–8710)	<0.05*
Lymphocyte (number/mm^3^)	1000–4000	1530 (1140–2050)	1800 (1350–2520)	0.162
Monocyte (number/mm^3^)	100–700	510 (390–700)	480 (340–790)	0.869
RDW	12.2–16.1	12.8 (12.175–13.6)	13.4 (13.1–14.8)	0.005*
Creatinine (mg/dL)	0.84–1.21	0.84 (0.76–0.98)	0.89 (0.65–1.12)	0.578
AST (U/L)	5–40	19.4 (14.7–26.2)	19.6 (15.7–49.5)	0.176
ALT (U/L)	7–56	18.1 (11.7–29.5)	17.5 (10.2–37)	0.940
GGT (U/L)	9–48	18 (12–32)	39 (30–104)	<0.05*
Total bilirubin (mg/dL)	0.1–1.2	0.41 (0.29–0.56)	0.55 (0.30–0.80)	0.051
Albumin (g/L)	34–54	45.12 (42–47)	37.46 (35.32–41.48)	<0.05*
CRP (mg/L)	0–10	2.55 (1.13–8.08)	71.42 (26.72–99.6)	<0.05*
Ferritin (ng/mL)	20–250	110.9 (44.5–170.2)	756.2 (237.1–1036)	<0.05*
IL-6 (pg/mL)	0–16.4	1.5 (1.5–1.5)	42.58 (11.92–101.8)	<0.05*
Procalcitonin (ng/mL)	0.1–0.49	0.025 (0.02–0.035)	0.138 (0.042–0.858)	<0.05*
N/L ratio	1–3	2.03 (1.37–3.39)	3.09 (1.87–5.64)	0.011*

**Figure 1:**
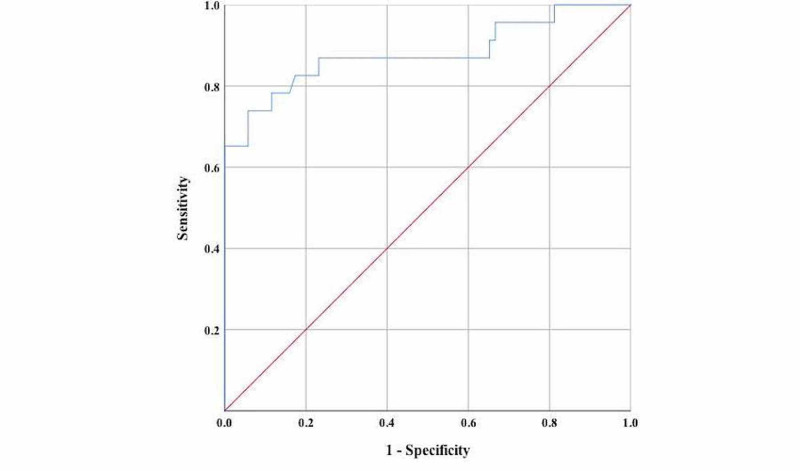
Receiver operating characteristic (ROC) curve for ferritin area under curve (AUC) = 0.880 (p<0.05); 95% CI = 0.780 – 0.980

A linear regression analysis in which age, WBC, neutrophil, GGT, albumin, CRP, ferritin, IL-6, procalcitonin, and N/L ratio were assigned as independent factors of disease severity revealed that the ferritin level was the only significant predictor of disease severity (β = 0.487, t = 2.993, p = 0.004). Receiver operating characteristic (ROC) curve analysis was performed for determining the best cut-off value of ferritin level for predicting disease severity. It was found that ferritin level ≥264.5 predicted the disease severity with a sensitivity of 73.9% and specificity of 94.2% (area under curve (AUC): 0.880, p < 0.05, Figure 1).

Overall mortality rate was 16.1% (Table 3). Of the deceased patients, 6.7% (n = 1) were aged in their 40s, 6.7% (n = 1) in their 50s, 53.3% (n = 8) in their 60s, and 33.3% (n = 5) in their 70s. Patients in the severe group had significantly higher mortality rate compared with those in the mild group (60.9% vs. 1.4%, p < 0.05)

**Table 3:** Mortality rates *p<0.05 Fisher’s exact test. MM, mild-moderate; SC, severe-critical

	Group MM (n=70) n (%)	Group SC (n=23) n (%)	P
Discharged	69 (98.6)	9 (39.1)	<0.05*
Exitus	1 (1.4)	14 (60.9)	

## Discussion

The present study showed that comorbidities, including diabetes, hypertension, heart failure, and coronary artery disease, may play important roles in disease severity. In addition, dyspnea was the most prominent symptom in patients with severe cases. Moreover, ferritin level was significantly higher in the severe group and was found to be the only significant predictor of disease severity in linear regression analysis.

Several studies have been conducted to elucidate the effects of COVID-19 on hemocytometric and biochemical parameters. In these studies it is clearly understood that SARS-CoV-2 infection has a high mortality rate, particularly among elderly patients with comorbidities [12,13]. The estimated increase in severity with age is reported in several cases, with reports that the mean age is between 50 and 60 years [14]. Liu et al. revealed that patients over 60 years tend to develop respiratory failure. This demonstrated that elderly patients with COVID-19 had more severe disease compared to younger patients [15]. The present study also found that elderly COVID-19 patients with ages in the 50s and 60s tended to have more severe disease than younger patients. Additionally, the fatality rate was higher in the elderly population (53.3% in patients in their 60s). The higher mortality rate in the elderly population might be explained by an increase in comorbidities with advancing age.

While the prevalence of diabetes among patients with COVID-19 varies across reports, Li et al. showed that the presence of diabetes was two-fold higher in severe cases than in mild cases, and that 9.7% of patients with COVID-19 had diabetes [16]. Another analysis demonstrated that 85.4% of severe COVID-19 patients had diabetes or cardiovascular diseases compared to the mild group [17]. According to Chinese data, the prevalence of diabetes in patients with severe COVID-19 is between 15%-25%, where it is two- to four-fold higher compared to non-critical patients [18,19]. Prevalence above 50% was also reported in the USA in patients admitted to ICU [20]. The present study also revealed a higher prevalence (17.4%) of diabetes in severe COVID-19 patients compared to their mild counterparts. The blood glucose level is thought to affect the immune system by increasing its susceptibility to SARS-CoV-2 infection and several infectious diseases as well [17].

Furthermore, hypertension is the most prevalent comorbidity among COVID-19 patients. It is reported with a prevalence of 17% and is two-fold higher in severe cases compared to mild ones [21]. A meta-analysis demonstrated that hypertension is rarer in discharged patients than in patients deceased from COVID-19. Also, a positive correlation was detected between hypertension and severity of the disease [22]. The present study revealed a three-fold higher prevalence of hypertension in patients with severe COVID-19 compared with patients with mild COVID-19. Moreover, HF and coronary artery disease (CAD) were more frequent in severe COVID-19 patients than in mild ones, and in those closely associated with the presence of diabetes and hypertension. Previous data confirmed the finding that cardiovascular diseases increase the severity of COVID-19, as well as mortality among patients with COVID-19 [23].

Despite finding that many laboratory and biochemical parameters were significantly different between the severe and mild groups, only CRP, IL-6, and ferritin levels were found to be pathologically elevated. CRP is a well-known biomarker of inflammation and is found elevated in 60.7% of patients with COVID-19. More severe cases demonstrated a more evident elevation in CRP levels compared to non-severe cases (81.5% vs 56.4%, respectively) [18]. Higher CRP levels are also linked to development of acute respiratory distress syndrome, higher troponin-T levels, and myocardial injury, which is observed in patients with severe COVID-19 [19,24,25]. We found increased CRP levels in severe cases of COVID-19 compared to mild ones. This can be assessed as a sign of intense inflammation. Additionally, another inflammatory marker for COVID-19 is elevated IL-6 in severe cases as seen in the present study. A retrospective cohort study in China reported that increased IL-6 levels were detected in non-survivors compared to survivors [6]. In addition, Chen et al. showed that 52% of patients with COVID-19 had elevated IL-6 values on admission to the hospital [12]. Increased IL-6 serum levels are closely related to raised risk of death. A progressive elevation of IL-6 during hospital stay has been demonstrated in non-survivors [6,13]. We also found increased levels of IL-6 in severe COVID-19 patients compared with mild patients.

Recent data have reported that patients with COVID-19 also have elevated levels of ferritin due to the inflammatory process. Hyperferritinemia has been accepted as an acute-phase reaction parameter that is used by clinicians to assess therapeutic response. In contrast, current research suggests that higher ferritin levels can be detected during an acute-phase response and may also play an important role in inflammation regarding development of a cytokine storm [9]. We found that serum ferritin level was significantly higher in severe cases. Similar to our study, a previous study also reported that elevated ferritin levels were observed in non-survivors [26]. Moreover, it was found that ferritin levels tend to increase with disease severity [6,27]. Furthermore, Zhou et al. revealed that hospital death rate was higher in patients with serum ferritin levels >300 ng/mL than in patients with serum ferritin levels ≤300 ng/mL [6]. Cao et al. demonstrated that a cut-off value of ferritin level ≥272.5 ng/mL predicted disease severity on admission with a sensitivity of 96%, and a specificity of 70% (AUC=0.873) [28]. Similarly, we also found that ferritin level ≥264.5 predicted disease severity with a sensitivity of 73.9% and specificity of 94.2% (AUC = 0.880). When all these findings were evaluated together, it was concluded that hyperferritinemia is an independent risk factor in COVID-19 patients and that it can also predict disease severity. There are two explanations that may define the importance of ferritin. According to Shoenfeld et al., the clinical course of severe COVID-19 patients mimics that of macrophage activating syndrome, which is characterized by elevated ferritin levels and the presence of a cytokine storm. The H-chain of ferritin activating macrophages is responsible for the increased secretion of inflammatory cytokines in patients with COVID-19 [29]. Another possible explanation could be that ferritin elevation might be how iron metabolism supports the immune system response to infecting microorganisms, including viral infections. Improved cellular metabolism and optimal iron levels among host cells are required for viral replication. Therefore, limiting the bioavailability of iron is key to disturbing the replication of the virus. Despite the underlying etiology, serum ferritin is mostly increased in patients with COVID-19. It would be useful to evaluate if serum ferritin can be used as a screening biomarker for the intensity of inflammation in patients with COVID-19 [10]. Nevertheless, ferritin can be considered to have a good ability to predict poor outcomes.

This study has several limitations. First, it is a single-centered retrospective study with a small sample size, which could restrict the generalizability of the results. Second, our findings cannot be generalized to all ethnicities because only Turkish people were included. Third, we reported only hospitalized cases. Patients who are being quarantined at home have not been considered.

## Conclusions

In conclusion, early analysis of ferritin levels in patients with COVID-19 might effectively define the severity of the disease. Therefore, ferritin holds a crucial role as a simple complementary tool for guiding the clinical decision and treatment. However, further studies are required to confirm these outcomes and to explain the precise pathological mechanisms.
